# ICTV Virus Taxonomy Profile: *Nodaviridae*

**DOI:** 10.1099/jgv.0.001170

**Published:** 2018-11-15

**Authors:** A. S. Sahul Hameed, A. S. Ninawe, T. Nakai, S. C. Chi, K. L. Johnson, ICTV Report Consortium

**Affiliations:** ^1^​ Aquatic Animal Health Laboratory, C. Abdul Hakeem College, Melvisharam 632509, Vellore Dt, TN, India; ^2^​ Advisor, Department of Biotechnology, Government of India, CGO Complex, New Delhi 110003, India; ^3^​ Graduate School, Hiroshima University, Japan; ^4^​ National Taiwan University, Taipei, Taiwan, ROC; ^5^​ Department of Biological Sciences, University of Texas at El Paso, Texas 79968, USA

**Keywords:** *Nodaviridae*, ICTV Report, taxonomy

## Abstract

The family *Nodaviridae* includes two genera, *Alphanodavirus* and *Betanodavirus*. The family name derives from the Japanese village of Nodamura where Nodamura virus was first isolated from *Culex tritaeniorhynchus* mosquitoes. Virions are non-enveloped and spherical in shape with icosahedral symmetry (T=3) and diameters ranging from 25 to 33 nm. The genome consists of two molecules of single-stranded positive-sense RNA: RNA1 and RNA2. The virion capsid consists of 180 protein subunits arranged on a T=3 surface lattice. Alphanodaviruses infect insects, whereas betanodaviruses are pathogens of fish. This is a summary of the International Committee on Taxonomy of Viruses (ICTV) Report on the taxonomy of the *Nodaviridae*, which is available at www.ictv.global/report/nodaviridae.

## Virion

**Fig. 1.**
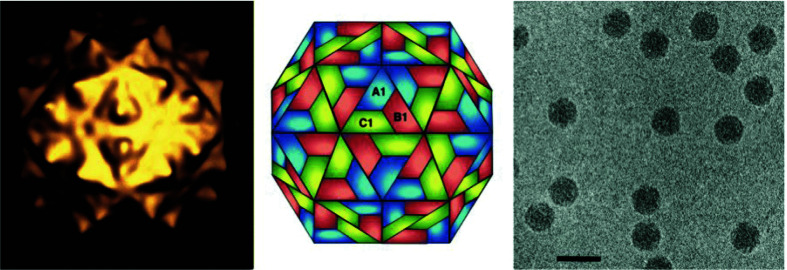
Flock House virus particles. (Left) Image reconstruction. (Centre) Schematic representation of a T=3 icosahedral lattice. A1, B1 and C1 indicate three different quasi-equivalent copies of the capsid protein. (Right) Cryo-electron micrograph; the bar represents 50 nm. (Courtesy of N. Olson and T. Baker.)

Virions are non-enveloped, roughly spherical in shape, 25–33 nm in diameter and have icosahedral symmetry (T=3) (Table 1, Fig. 1) [1]. Electron microscopy of negatively stained betanodaviruses shows surface projections; these are not observed in alphanodaviruses. Virion buoyant density in CsCl ranges from 1.30 to 1.36 g cm^−3^. Virions are stable to pH values ranging from 2 to 9 and are resistant to heating at 56 °C for 30 min.

**Table 1.** Characteristics of the family *Nodaviridae*

Typical member:	Striped jack nervous necrosis virus (RNA1: AB056571; RNA2: AB056572), species *Striped jack nervous necrosis virus*, genus *Betanodavirus*
Virion	Non-enveloped spherical particles, 25–33 nm in diameter, with or without surface projections
Genome	Bi-partite single-stranded positive-sense RNA of 3.1 kb (RNA1) and 1.4 kb (RNA2) with 5′-terminal caps but without poly(A) tails
Replication	Cytoplasmic within virus-induced invaginations on the outer mitochondrial membrane
Translation	From capped genomic and subgenomic RNAs
Host range	Natural hosts are insects (*Alphanodavirus*) or fish (*Betanodavirus*)
Taxonomy	Two genera, each including four or more species

## Genome

The genome of nodaviruses consists of two molecules of positive-sense single-stranded RNA; RNA1 (3.1 kb) encoding protein A, an RNA-dependent RNA polymerase of about 112 kDa (983–1014 amino acids), and RNA2 (1.4 kb) encoding protein α, the capsid protein precursor. Both RNA molecules are encapsidated in the same virus particle, and both are required for infectivity. Both molecules are capped at their 5′-ends and lack poly(A) tails at their 3′-ends [2].

## Replication

**Fig. 2.**
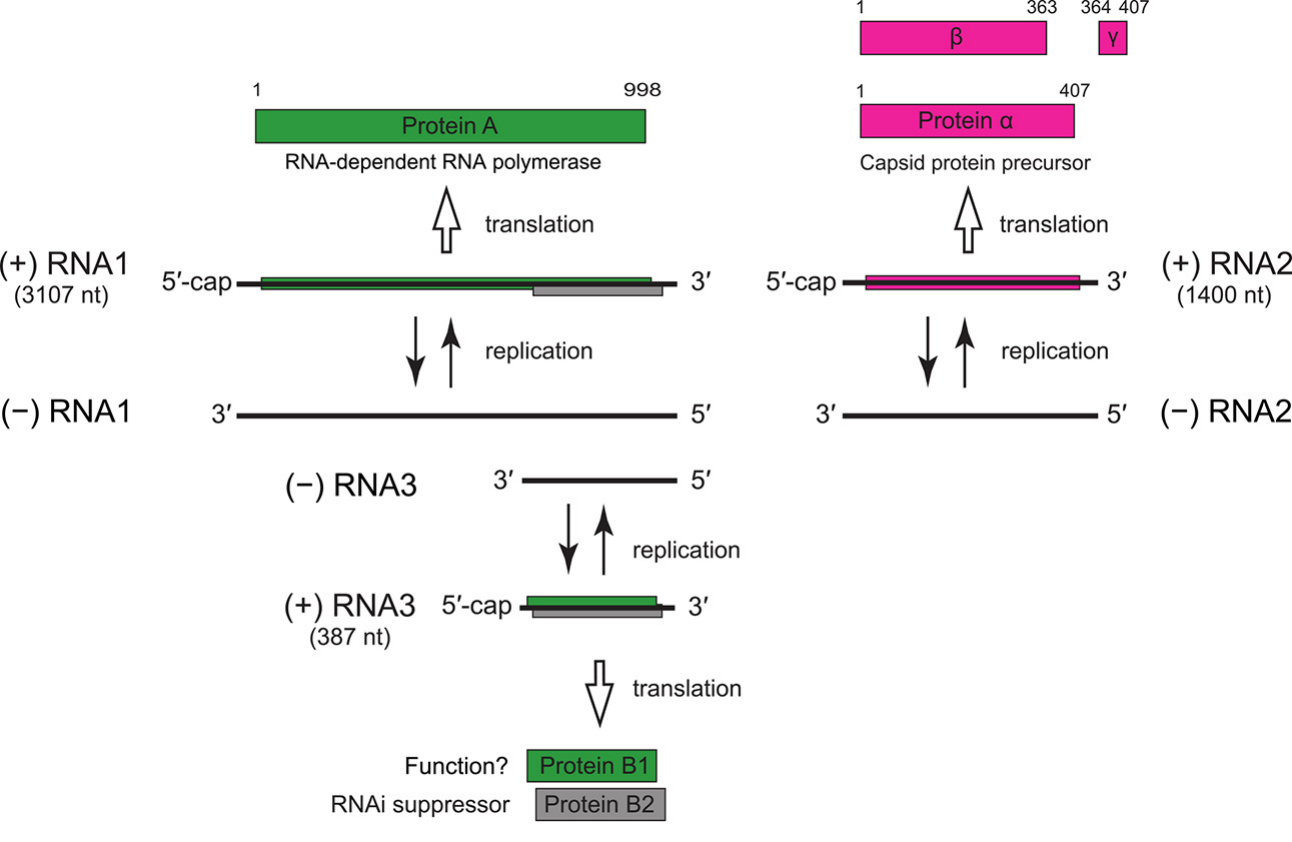
Flock House virus (*Alphanodavirus*) genome organization and replication strategy.

Virus replication is cytoplasmic. Infected cells contain single-stranded RNAs corresponding to RNA1 and RNA2, as well as the subgenomic RNA3 (387 nucleotides), which derives from the 3′-terminus of RNA1 and is not packaged into virions (Fig. 2). In addition to RNA-dependent RNA polymerase activity, protein A binds to and drives invagination of the outer mitochondirial membrane to provide the compartment where RNA replication occurs. RNA3 encodes either one or two proteins; protein B2 (11 kDa) is encoded by all nodaviruses in a reading frame overlapping that of protein A, and is a suppressor of RNA interference. Some nodaviruses also express protein B1 (11 kDa), which corresponds to the C-terminal region of protein A and is of unknown function. Maturation of non-infectious provirions involves the autocatalytic cleavage of protein α into proteins β (39 kDa) and γ (4 kDa).

## Pathogenicity

Alphanodavirus infection results in the stunting, paralysis and death of the insect host. The infection of fish by betanodaviruses such as striped jack nervous necrosis virus or red-spotted grouper nervous necrosis virus causes neural necrosis, encephalopathy or retinopathy and is associated with behavioural abnormalities and high mortality, posing significant problems for marine aquaculture [3, 4].

## Taxonomy

The members of the two genera in this family infect insects (*Alphanodavirus*) [5] or fish (*Betanodavirus*) [6]. Different species encode capsid proteins that differ at >20 % of nucleotide or >13 % of amino acid positions (*Alphanodavirus*) or >15 % of nucleotide and >12 % of amino acid positions (*Betanodavirus*). There is limited sequence identity between the capsid proteins of members of different genera, or with those of a number of unclassified nodaviruses isolated from prawns, nematodes and insects.

## Resources

Full ICTV Online (10th) Report: www.ictv.global/report/nodaviridae.
